# Effect of Vitamins A, C, and E Supplementation in the Treatment of Metabolic Syndrome in Albino Rats

**DOI:** 10.1155/2012/678582

**Published:** 2012-08-16

**Authors:** L. S. Bilbis, S. A. Muhammad, Y. Saidu, Y. Adamu

**Affiliations:** ^1^Biochemistry Department, Usmanu Danfodiyo University, PMB 2346, Sokoto, Nigeria; ^2^Faculty of Veterinary Medicine, Usmanu Danfodiyo University, PMB 2346, Sokoto, Nigeria

## Abstract

Obesity and metabolic syndrome increase the risk of cardiovascular morbidity and mortality. Oxidative stress seems to be involved in the path physiology of cardiovascular complications of metabolic syndrome. In this study we investigated the effects of vitamins A, C, and E in the management of metabolic syndrome traits condition in albino rats fed with high salt diet. The rats were placed on 8% NaCl diet for 5 weeks and then supplemented with these vitamins for additional 4 weeks in the presence of salt diet. Supplementation with vitamins significantly (*P* < 0.01 ) decreased blood pressure of the rats as compared with the control. Supplementation also significantly (*P* < 0.05) reduced serum total cholesterol, triglyceride, low-density lipoprotein cholesterol, and very-low-density lipoprotein cholesterol and increased high-density lipoprotein cholesterol, and total antioxidant status as compared with untreated group. The percentage protection of the supplemented groups against atherogenesis indicated 55.50 ± 3.75%. Percentage weight gain indicated significant positive correlation with triglyceride, insulin resistance, and malondialdehyde while total antioxidant status and nitric oxide showed significant negative correlation. Salt diet significantly (*P* < 0.05) induced features of metabolic syndrome. The result, therefore, indicated strong relationship between obesity and metabolic syndrome and underscores the role of these vitamins in the management of metabolic syndrome.

## 1. Introduction

Metabolic syndrome, a constellation of cardiovascular risk factors that is characterized by hypertension, hyperglycemia, insulin resistance, dyslipidaemia, and abdominal obesity, is associated with increased risk of developing cardiovascular diseases [1–4]. High blood pressure is considered one of the key features of metabolic syndrome [5]. The increasing prevalence of metabolic syndrome is due to rising number of people who are obese and inactive [6, 7]. It is important to emphasize that obesity increases the likelihood of an individual to develop insulin resistance [8].

 The cluster of risk factors of metabolic syndrome is responsible for cardiovascular morbidity among overweight, obese, and type 2 diabetic subjects [9]. Approximately 1 adult in 4 or 5, depending on the country, shows features of the syndrome. In the category over 50 years of age, it affects more than 40% of the population in the United States and nearly 30% in Europe [10]. There is evidence of increasing incidence and prevalent of the metabolic syndrome in sub-Saharan Africans [11, 12].

However, the establishment of hypertension as a component of the metabolic syndrome has given better insight into the condition, known to be complex and multifactorial [13] and therefore allowed for earlier detection and treatment. In fact, hypertension affects up to 85% of patients with metabolic syndrome [14].

Several studies indicated the role of oxidative stress in the pathogenesis of hypertension [15]. Oxidative stress may account for endothelial dysfunction, but it is unknown whether this abnormality is a primary event or a consequence of increased blood pressure [16]. Studies have also shown that exogenous administration of antioxidants improved the vascular function and reduced blood pressure in animal models [17] and in human hypertension [18, 19]. Thus, the study aimed to investigate the effects of vitamins A, C, and E on salt-induced metabolic syndrome traits in albino rats.

## 2. Methods

### 2.1. Chemicals and Reagents

Analytical graded chemicals and reagents were used for this research. Vitamin A capsule was purchased from Pharco Pharmaceuticals Industries, Alexandria, Egypt; vitamin C was sourced from Nemen pharmaceutical industries, Enugu, Nigeria, and vitamin E (ENAT 400) capsule from Mega Lifesciences Ltd, Thailand.

### 2.2. Experimental Animals

Wistar rats weighing between 150 and 180 g were purchased from Faculty of Veterinary Medicine, Usmanu Danfodiyo University, Sokoto, Nigeria and were allowed to acclimatize for two weeks before the commencement of the experiment. The animals were placed into 6 groups of 5 rats each and were fed pelletized growers' feed (Vital feed, Jos, Nigeria). The animals were also allowed access to clean water *ad libitum *throughout the experimental period. The experimental protocol was approved by the Ethical Committee of the Usmanu Danfodiyo University, Sokoto, Nigeria.

### 2.3. Preparation of Hypertensive Rats/Induction of Metabolic Syndrome Trait

The rats were placed on high salt (8% NaCl) diet for 5 weeks *ad libitum *and supplementation in the presence of the challenging agent for additional 4 weeks. 

### 2.4. Measurement of Blood Pressure

The blood pressure was monitored on weekly basis by tail-cuff method using noninvasive Ugo Basile, series 58500 Blood Pressure Recorder. Average of four readings was taken for each rat and the temperature of the rats was monitored throughout the measurement period. 

### 2.5. Preparation of Supplements

Capsules of vitamin A and E were cut, open, and emptied into separate clean containers. Palm olein was added to prepare a suspension containing 7.5 mg of the vitamin A and 67 mg of vitamin E in 1 mL. Each tablet (100 mg) of vitamin C was crushed and dissolved in 2 mL of distilled water to obtain 50 mg/mL suspension. All the supplements were prepared just prior to administration.

### 2.6. Grouping of Animals and Treatment

The animals were randomly divided into six groups of 5 rats each:

Group I: normal untreated (1 mg/kg of palm olein);
Group II: hypertensive control (1 mg/kg of palm olein);
Group III: salt-loaded treated with 100 mg/kg of vitamin C;
Group IV: salt-loaded treated with 6 mg/kg of vitamin A;
Group V: salt-loaded treated with 60 mg/kg of vitamin E;
Group VI: salt-loaded treated with 6 mg/kg of vitamin A, 100 mg/kg of vitamin C, and 60 mg/kg of vitamin E.

The appropriate dosages of the supplements were administered orally to the animals according to their body weight once daily by intubation using intravenous cannula tube for 4 weeks. Twenty-four hours after the last treatment, the animals were anaesthetized with chloroform vapour and fasting blood samples were collected through cardiac puncture into labelled tubes for biochemical analyses. Weight changes of the rats were monitored throughout the experimental period.

### 2.7. Biochemical Estimations

The fasting serum glucose level was estimated by the glucose oxidase method [20]. Estimation of serum total cholesterol [21], triglyceride [22], and high-density lipoprotein cholesterol [23] were done by enzymatic method. Serum low-density lipoprotein cholesterol and very-low-density lipoprotein cholesterol were calculated by the formula of Friedewald et al. [24]. Atherogenic index was calculated as the ratio of LDL cholesterol to HDL-cholesterol [25].

Colorimetric method was used to estimate vitamin E [26], vitamin C [27], total antioxidant status [28], and tissue malondialdehyde [29] while Cayman's Assay Kit was used to assay superoxide dismutase, catalase, glutathione peroxidase, and total nitric oxide. Insulin was estimated by SPI bio rat insulin enzyme immunoassay kit.

Insulin resistance index was calculated by homeostasis model assessment-insulin resistance (HOMA-IR) [30].

(1) $$\begin{array}{c} \text{HOMA-IR}=\frac{\text{Fasting}\;\text{glucose}\;\left(\text{mmol}/\text{L}\right)\times \text{Fasting}\;\text{insulin}\;\left(\mu \text{U}/\text{mL}\right)}{22.5}. \end{array}$$

Percentage protection against atherogenesis was calculated using the following equation:

(2) $$\begin{array}{c} \frac{\text{Atherogenic}\;\text{index}\;\left(\text{AI}\right)\;\text{of}\;\text{hypertensive}\;\text{control}\;\left(\text{HC}\right)-\text{AI}\;\text{of}\;\text{treated}\;\text{group}}{\text{Atherogenic}\;\text{index}\;\left(\text{AI}\right)\;\text{of}\;\text{hypertensive}\;\text{control}\;\left(\text{HC}\right)}\times 100. \end{array}$$

### 2.8. Statistical Analysis

Values are expressed as mean ± standard deviation for 5 rats in each group. The biochemical parameters were analysed statistically using one way analysis of variance (ANOVA), followed by Dunnett's multiple comparison test using GraphPad Instat software. Differences were considered significant when *P* < 0.05.

## 3. Results

**Figure 1:**
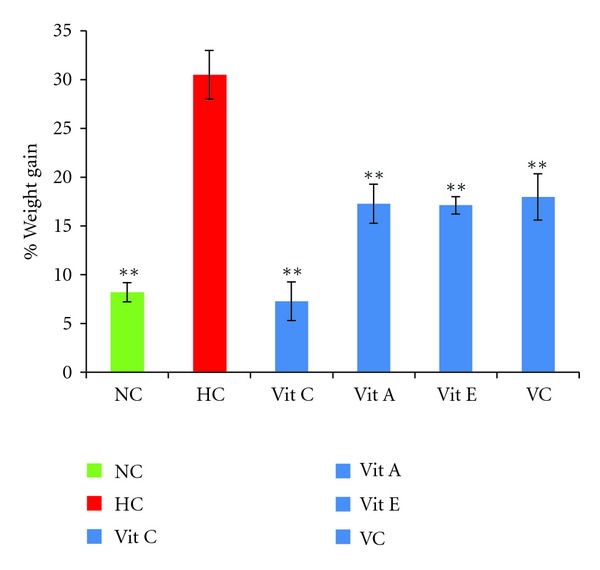
Percentage weight gain of salt-loaded rats supplemented with antioxidant vitamins. NC—normortensive control, HC—hypertensive control, Vit C—salt loaded treated with vitamin C, Vit A—salt loaded treated with vitamin A, Vit E—salt loaded treated with vitamin E, and VC—salt loaded treated with vitamins combined. *P < 0.01 when compared with HC.

The % weight gain of rats (Figure 1) indicated salt-loaded untreated control gain more weight (30.48%) than the treated groups and normotensive control. Supplementation significantly (*P* < 0.01) decreased the % weight gain of the supplemented groups as compared to the hypertensive control. 

**Figure 2:**
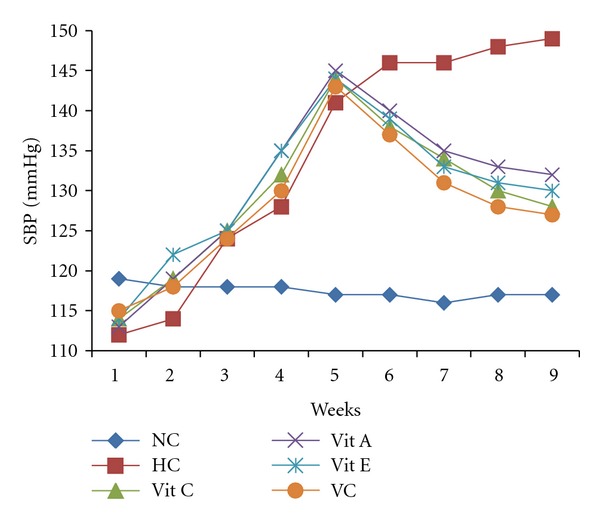
Effect of salt diet and antioxidant supplementation on systolic blood pressure of salt-loaded rat. week 1–5: salt diet only, week 6–9: -salt diet plus supplements, NC—normotensive, HC—hypertensive control, Vit C—salt loaded treated with vitamin C, Vit A—salt loaded treated with vitamin A, Vit E—salt loaded treated with vitamin E, and VC—salt loaded treated with vitamins combined.

The effect of supplementation on systolic blood pressure (SBP) is presented in Figure 2. Salt-loading increased SBP significantly (*P* < 0.01) as compared to the normotensive control and supplementation with vitamins decreased significantly (*P* < 0.01) the systolic blood pressure as compared with hypertensive control. 

Effect of supplementation on glucose, insulin, and insulin resistance is presented in Table 1. The supplementation significantly (*P* < 0.01) decreased serum glucose, insulin, and insulin resistance as compared with hypertensive control.

Effect of vitamins A, C, and E on serum lipid profile and AI is presented in Table 2. The result indicated significantly (*P* < 0.01) decreased serum TC, TG, LDL-C, VLDL-C, and AI while HDL-C increased significantly (*P* < 0.05) as compared with hypertensive control.

**Figure 3:**
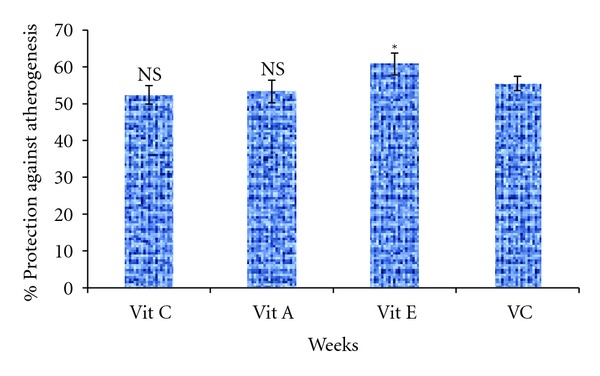
Percentage protection against atherogenesis of supplemented groups. Vit C—salt loaded treated with vitamin C, Vit A—salt loaded treated with vitamin A, Vit E—salt loaded treated with vitamin E, VC—salt loaded treated with vitamins combined. *P < 0.05, and NS—not significant when compared with VC.

The % protection against atherogenesis is presented in Figure 3. The supplementation significantly (*P* < 0.05) increased protection of the group supplemented with vitamin E (60.79%) as compared to vitamin combined group (55.50%) while vitamins A (53.30%) and C (52.42%) showed no significant difference in the protection. 

Effect of supplementation on vitamins C and E, total antioxidant status and malondialdehyde is presented in Table 3. Supplementation significantly (*P* < 0.01) decreased the level of tissue MDA while total antioxidant status increased significantly (*P* < 0.01) as compared with hypertensive control. The vitamin C and vitamin E levels also increased significantly (*P* < 0.05) except for the group that received vitamin A that showed no significant (*P* > 0.05) difference as compared with hypertensive control. The serum vitamin E level of the vitamin C supplemented group does not also differed significantly (*P* > 0.05) as compared with hypertensive control. 

The result of effect of supplementation on antioxidant enzymes and nitric oxide is presented in Table 4. The result indicated that supplementation increased significantly the activities of catalase, glutathione peroxidase, and superoxide dismutase as compared with hypertensive control. The level of nitric oxide also increased significantly (*P* < 0.01) as compared with hypertensive control.

 The correlation between % weight increase and metabolic syndrome markers is presented in Table 5. The result indicated significant positive correlation between % weight gain and TG, HOMA-IR, MDA, AI while NO and TAS showed significant negative correlation. 

## 4. Discussion

Metabolic syndrome is increasingly recognized as an independent predictor of cardiovascular disease in hypertension [4] and in the context of global cardiovascular risk, metabolic syndrome is indeed a high-risk condition, involving three or more risk factors, often organ damage and diabetes [10]. Metabolic syndrome-like condition was induced in experimental albino rats by placing the rats on 8% salt diet for 5 weeks and salt-loaded diet plus supplementation for additional 4 weeks. The salt-loading increased the blood pressure of the rats and the supplementation with antioxidant vitamins A, C, and E significantly decreased the blood pressure of the rats as compared with hypertensive group. Thus, the role of antioxidants in lowering the blood pressure could be attributed to their ability in scavenging free radical as reactive oxygen species seems to play significant role in the path physiology of hypertension [31]. 

There is strong relationship between hypertension and dyslipidemia and both may add up to increase the susceptibility to the development of coronary heart disease [32]. An elevated serum total cholesterol, triglyceride, low-density lipoprotein cholesterol, very-low-density lipoprotein cholesterol, and decreased high-density lipoprotein cholesterol concentration observed in salt-loaded untreated rats as compared to the supplemented rats in this study is similar to the findings of [33, 34] on hypertension. Supplementation with antioxidant vitamins significantly decreased serum total cholesterol, triglyceride, low-density lipoprotein cholesterol, and very-low-density lipoprotein cholesterol and increased high-density lipoprotein cholesterol level as compared with the hypertensive control. 

Several studies have found variable degrees of correlations between lipid parameters with measures of insulin resistance [35, 36] and indicated that insulin resistance/hyperinsulinemia, a hallmark of the metabolic syndrome, is a predictor of ischemic heart disease in the population at large [37] and in patients with type II diabetes [38]. 

Endothelial dysfunction is observed in most rat models of hypertension [39, 40]. Oxidative stress has been suggested to contribute to insulin resistance [41] and plays a critical role in the pathogenesis of endothelial dysfunction [42, 43]. The significant decreased in the levels of malondialdehyde, glucose, insulin, and insulin resistance and increased in the levels of vitamins C and E, total antioxidant status, catalase, superoxide dismutase, glutathione peroxidase, and nitric oxide in the supplemented groups as compared with hypertensive control indicated the role of oxidative stress in hypertension, insulin resistance, and endothelial dysfunction in this model. In this regard, the decreased nitric oxide level observed in hypertensive control might indeed reflect the impaired nitric oxide bioavailability. The possible link between insulin resistance and endothelial dysfunction is that binding of insulin to its receptor stimulates the production of nitric oxide at the endothelial level [44], and since nitric oxide constitutes one of the major vasodilator, the defect in insulin signalling pathway caused by insulin resistance appears to be closely associated with endothelial dysfunction.

However, a direct consequence of hypertension, insulin resistance, dyslipidemia, and endothelial dysfunction may enhance oxidative stress which could constitute the starting point for cardiovascular complications associated with the metabolic syndrome. The possible mechanism of how high salt diet induces metabolic syndrome responses is that salt does not only increase the blood pressure but also decreases insulin sensitivity in Dahl salt-sensitive rats [45]. Salt-induced insulin resistance might be attributable to the overproduction of reactive oxygen species, and it has been shown that not only adipokines released from visceral fat but also salt can induce insulin resistance in the muscles and adipose tissues through oxidative stress [46]. This is evidenced in our study by increased level of malondialdehyde, glucose, triglyceride, and insulin and decreased antioxidant activities in hypertensive control as compared with the supplemented groups.

The improved endothelial function and insulin sensitivity observed in the supplemented groups confirms the role of antioxidant vitamins in the management of metabolic syndrome. Thus, the exact molecular mechanisms underlying antioxidant effects of these vitamins on insulin sensitivity and endothelial function were not fully assessed in this model but could be attributed to their role in inhibiting NADPH oxidase activity, scavenging free radical, and stimulating the activity of nitric oxide synthase. Studies have shown that vitamins C and E [47] and vitamin C [48] can stimulate the activity of endothelial nitric oxide synthase by increasing the intracellular availability of the endothelial nitric oxide synthase cofactor tetrahydrobiopterin, which could further increase nitric oxide synthesis.

The significant positive correlation between % weight increase and triglyceride, insulin resistance, malondialdehyde, and atherogenic index and significant negative correlation between % weight increase and total antioxidant status and nitric oxide further confirmed the role of oxidative stress as the aetiology of cardiovascular complications of hypertension in particular and metabolic syndrome in general and underscores the role of antioxidant supplementation as the preventive and management strategies for the complications associated with metabolic syndrome. 

This study also provides evidence that metabolic syndrome may be useful as an integrating index of the overall burden imposed by metabolic factors on the cardiovascular system in hypertension.

## 5. Conclusion

The study indicated that salt loading may play an important role in oxidative stress, hypertension, dyslipidemia, insulin resistance, obesity, and endothelial dysfunction, some of which are indicators of metabolic syndrome. The results further suggest that supplementation may be used as preventive and management strategies for the cardiovascular complications of hypertension, diabetes and metabolic syndrome.

## Figures and Tables

**Table 1:** Effect of vitamins A, C, and E on glucose, insulin, and insulin resistance.

Group	Glucose (mmol/L)	Insulin (*μ*U/mL)	HOMA-IR
I	4.28 ± 0.31	2.98 ± 1.42	0.56 ± 0.23
II	6.50 ± 0.83^c^	17.01 ± 4.04^c^	4.81 ± 0.68^c^
III	5.28 ± 0.84^b,d^	4.53 ± 2.38^a^	1.06 ± 0.43^a^
IV	4.89 ± 0.42^a^	3.96 ± 1.48^a^	0.84 ± 0.26^a^
V	4.75 ± 0.30^a^	5.09 ± 1.07^a^	1.07 ± 0.19^a^
VI	4.83 ± 0.43^a^	5.40 ± 1.33^a^	1.14 ± 0.22^a^

HOMA-IR: homeostasis model assessment-insulin resistance, I: normotensive control, II: hypertensive control, III: group treated with vit C, IV: group treated with vit A, V: group treated with vit E, and VI: group treated with all the vitamins. Values are expressed as mean ± SD; *n* = 5. ^a^ *P* < 0.01 when compared with group II, ^b^ *P* < 0.05 when compared with group II, ^c^ *P* < 0.01 when compared with group I, and ^d^ *P* < 0.05 when compared with group I by Dunnette's multiple comparison test.

**Table 2:** Effect of antioxidant vitamins on lipid profile and atherogenic index.

Group	TC (mg/dL)	TG (mg/dL)	HDL-C (mg/dL)	LDL-C (mg/dL)	VLDL-C (mg/dL)	AI
I	78.08 ± 4.03	63.89 ± 5.17	40.44 ± 5.30	24.53 ± 4.85	12.77 ± 1.03	0.61 ± 0.16
II	123.64 ± 7.47^c^	121.10 ± 12.14^c^	30.36 ± 2.23	69.05 ± 5.83^c^	24.21 ± 2.42^c^	2.27 ± 0.20^c^
III	104.84 ± 6.96^a, c^	76.08 ± 7.69^ a^	43.72 ± 9.82	45.80 ± 7.62^a, c^	15.21 ± 1.54^a^	1.08 ± 0.26^a, d^
IV	101.52 ± 9.29^a, c^	78.75 ± 3.19^ a^	41.79 ± 6.08^ b^	43.98 ± 5.26^a, c^	15.74 ± 0.63^a^	1.06 ± 0.18^a, d^
V	97.21 ± 8.01^a, c^	81.87 ± 13.86^ a, d^	43.97 ± 11.46^ b^	37.07 ± 9.93^a, d^	16.16 ± 2.75^a, d^	0.89 ± 0.34^a^
VI	101.45 ± 6.22^a, c^	86.12 ± 8.52^a, c^	42.79 ± 2.86^ b^	42.44 ± 4.27^a, c^	17.22 ± 1.70^a, c^	1.01 ± 0.12^a, d^

TC: total cholesterol, TG: triglyceride, HDL-C: high-density lipoprotein cholesterol, LDL-C: low-density lipoprotein cholesterol, VLDL-C: very-low-density lipoprotein cholesterol, AI: atherogenic index, I: normotensive control, II: hypertensive control, III: group treated with vitamin C, IV: group treated with vitamin A, V: group treated with vitamin E, and VI: group treated with all the vitamins. Values are expressed as mean ± SD; *n* = 5. ^a^ *P* < 0.01 when compared with group II, ^b^ *P* < 0.05 when compared with group II, ^c^ *P* < 0.01 when compared with group I, and ^d^ *P* < 0.05 when compared with group I by Dunnette's multiple comparison test.

**Table 3:** Effect of antioxidant supplementation on antioxidant indices and lipid peroxidation.

Group	Vitamin C	Vitamin E	TAS	MDA
	(mg/dL)	(mg/dL)	(mmol/L)	(nmoL/mg tissue)
I	0.95 ± 0.15	0.73 ± 0.32	1.64 ± 0.24	0.382 ± 0.04
II	0.51 ± 0.05^c^	0.47 ± 0.01	0.70 ± 0.17^c^	1.210 ± 0.22^c^
III	0.74 ± 0.17^b,d^	0.63 ± 0.06	1.33 ± 0.27^a^	0.402 ± 0.08^a^
IV	0.66 ± 0.03^c^	0.63 ± 0.05	1.16 ± 0.22^b,d^	0.425 ± 0.22^a^
V	0.74 ± 0.08^b^	0.77 ± 0.15^b^	1.33 ± 0.28^a^	0.423 ± 0.10^a^
VI	0.81 ± 0.17^a^	0.78 ± 0.12^b^	1.42 ± 0.34^a^	0.432 ± 0.06^a^

TAS-total antioxidant status, MDA-malondialdehyde, I: normotensive control, II: hypertensive control, III: group treated with vit C, IV: group treated with vit A, V: group treated with vit E, and VI: group treated with all the vitamins. Values are expressed as mean ± SD; *n* = 5. ^a^ *P* < 0.01 when compared with group II, ^b^ *P* < 0.05 when compared with group II, ^c^ *P* < 0.01 when compared with group I, and ^d^ *P* < 0.05 when compared with group I by Dunnette's multiple comparison test.

**Table 4:** Effect of supplementation on antioxidant enzymes and nitric oxide.

Group	Catalase	GPx	SOD	Nitric oxide
	(nmol/min/mL)	(nmol/min/mL)	(U/mL)	(*μ*M)
I	26.27 ± 4.47	95.76 ± 9.75	5.21 ± 1.46	28.14 ± 3.85
II	14.52 ± 3.16^c^	28.01 ± 7.26^c^	2.86 ± 0.62^c^	17.70 ± 3.07^c^
III	23.76 ± 5.84^a^	73.34 ± 11.62^a,d^	5.49 ± 1.06^a^	28.59 ± 4.86^a^
IV	21.48 ± 3.70	61.88 ± 10.44^a,c^	4.93 ± 0.83^b^	29.33 ± 4.30^a^
V	24.65 ± 3.19^a^	78.19 ± 10.40^a^	6.38 ± 0.58^a^	26.96 ± 4.87^a^
VI	22.16 ± 4.14^b^	72.58 ± 12.86^a,c^	6.01 ± 1.28^a^	22.81 ± 3.51

GPx: glutathione peroxidase, SOD: superoxide dismutase, I: normotensive control, II: hypertensive control, III: group treated with vit C, IV: group treated with vit A, V: group treated with vit E, and VI: group treated with all the vitamins. Values are expressed as mean ± SD; *n* = 5. ^a^ *P* < 0.01 when compared with group II, ^b^ *P* < 0.05 when compared with group II, ^c^ *P* < 0.01 when compared with group I, and ^d^ *P* < 0.05 when compared with group I by Dunnette's multiple comparison test.

**Table 5:** Correlation coefficient (*r*) of % wt increase against metabolic syndrome markers.

% weight increase	Correlation coefficient (*r*)	*P* value
TG	0.930	*P* = 0.007
HOMA-IR	0.849	*P* = 0.032
Nitric oxide	−0.844	*P* = 0.034
TAS	−0.858	*P* = 0.028
MDA	0.850	*P* = 0.032
AI	0.843	*P* = 0.035

HOMA-IR: homeostasis model assessment-insulin resistance, TG: triglyceride, AI: atherogenic index, TAS: total antioxidant status, and MDA: malondialdehyde.
